# Sirolimus reduces the risk of pneumothorax recurrence in patients with lymphangioleiomyomatosis: a historical prospective self-controlled study

**DOI:** 10.1186/s13023-022-02418-2

**Published:** 2022-07-08

**Authors:** Chongsheng Cheng, Wenshuai Xu, Yani Wang, Tengyue Zhang, Luning Yang, Wangji Zhou, Danjing Hu, Yanli Yang, Xinlun Tian, Kai-Feng Xu

**Affiliations:** State Key Laboratory of Complex Severe and Rare Diseases, Department of Pulmonary and Critical Care Medicine, Peking Union Medical College Hospital, Peking Union Medical College, Chinese Academy of Medical Sciences, Beijing, China

**Keywords:** Lymphangioleiomyomatosis, Pneumothorax, Recurrence, Sirolimus

## Abstract

**Background:**

Spontaneous pneumothorax has a high incidence and high rate of recurrence in patients with lymphangioleiomyomatosis (LAM). The risk factors for pneumothorax and the effects of sirolimus on pneumothorax in patients with LAM are unknown. In our study, multivariate logistic regression was applied to a cross-sectional cohort to investigate factors associated with pneumothorax in LAM patients. Kaplan–Meier analysis was applied in the historical prospective self-controlled study to determine whether sirolimus reduces the risk of pneumothorax recurrence in patients with LAM.

**Results:**

Of the 399 patients registered with LAM-CHINA at our center between May 10, 2017 and August 31, 2020, 142 had a history of pneumothorax at registration. High CT grade and age at presentation ≤ 35 years were associated with a higher risk of pneumothorax in patients with LAM. Postmenopausal status was correlated with a lower risk of pneumothorax. In the historical prospective self-controlled study, the 5-year probability of pneumothorax recurrence was 80% lower in the sirolimus group than in the control group (hazard ratio for pneumothorax recurrence, 0.20; 95% CI, 0.14 to 0.30, *P* < 0.001 by log-rank test).

**Conclusion:**

Sirolimus reduced the risk of pneumothorax recurrence in LAM patients.

## Background

Lymphangioleiomyomatosis (LAM) is a rare, devastating lung disease characterized by diffuse cystic changes, and it almost exclusively occurs in women. LAM can be divided into two categories: sporadic LAM without a genetic background and hereditary tuberous sclerosis complex-associated LAM (TSC-LAM) [1]. According to several observational studies that included more than 100 LAM patients [2–7], spontaneous pneumothorax occurs frequently in LAM patients, with a prevalence rate of 42.5% to 69%. Over 70% of LAM patients with a pneumothorax history experience at least one recurrence, and even 10 or more recurrences were observed in some cases [3, 5, 6]. Repeated pneumothorax may cause acute exacerbation of LAM and put a considerable financial or psychological burden on patients. Due to the high recurrence rate of pneumothorax in patients with LAM, pleurodesis is suggested for patients after the first episode of pneumothorax [8]. However, pleurodesis is an invasive procedure that is associated with an increased risk of bleeding during lung transplantation [3]. Effective alternative treatment options are needed.

Sirolimus, a mammalian target of rapamycin inhibitor, has been shown to decelerate pulmonary function loss and improve the quality of life in LAM patients [9]. Interestingly, a case series [10] found that the frequency of pneumothorax decreased after the application of sirolimus in LAM patients with recurrent pneumothorax. However, there were no significant differences in the probability of pneumothorax recurrence between LAM patients who initiated sirolimus postoperatively and those who did not [10]. Hence, the effect of sirolimus on pneumothorax in patients with LAM is unclear.

This report aimed to investigate the factors associated with pneumothorax and to determine whether sirolimus reduces the risk of pneumothorax recurrence in patients with LAM.

## Methods

### Patients and data collection

For the cross-sectional analysis, patients registered with LAM-CHINA (NCT 03193892) at our center between May 10, 2017 and August 31, 2020 were reviewed. All patients who met the American Thoracic Society (ATS)/Japanese Respiratory Society (JRS) diagnostic criteria of definite LAM were included. Patients with a history of iatrogenic pneumothorax were excluded. Patient diagnostic category, demographic data, current and past medical history, serum vascular endothelial growth factor-D (VEGF-D), pulmonary function test, 6-min walk test (6MWT), and CT images of the chest, abdomen, and pelvis were collected on the date of registration. The CT grade was assessed as described [11]. For patients with a pneumothorax history, postmenopausal status was defined as having reached menopause before their first pneumothorax.

### Follow-up

In the historical prospective self-controlled study, LAM patients were followed up until August 26, 2021. Pneumothorax data from the initial presentation of LAM to the last follow-up were gathered by medical record review and telephone inquiry. The diagnosis of pneumothorax was based on chest imaging. Data on the date, side, and triggers of pneumothorax and whether pleurodesis was performed were collected and confirmed.

### Data analysis

Prism 9.0 (GraphPad Software, San Diego), SPSS Statistics 23 (SPSS Inc, Chicago), and JMP 16 (SAS Software, USA) were used for the statistical analysis of the data. The statistically significant *P*-value threshold was 0.05 (two-tailed).

Patients were observed from the initial symptoms of LAM to the last follow-up to calculate the overall incidence of pneumothorax. We assumed that patients with LAM were continuously exposed to the risk of pneumothorax from the moment of onset, regardless of the occurrence of pneumothorax. Pneumothorax recurrence within two weeks was considered an unresolved event rather than a new event. However, when pneumothorax was the first symptom attributed to LAM, it was included in the numerator of the calculation. The incidence, in the numerator, was defined as the number of pneumothorax occurrences during the observation period, and the denominator was the sum of the follow-up person-years.

To explore the factors associated with the occurrence of pneumothorax in patients with LAM at registration, a multivariate logistic regression was performed. After excluding variables with potential collinearity, variables with significant differences in univariate analysis were included. Considering the collinearity between variables such as age at registration, age at presentation, age at diagnosis, and duration of course, we included the age at presentation in the multivariate logistic regression model because pneumothorax did not occur before the initial presentation of LAM. Pulmonary function-related parameters had missing values in 37% of patients with pneumothorax history, and these parameters were not entered into the regression model.

**Fig. 1:**
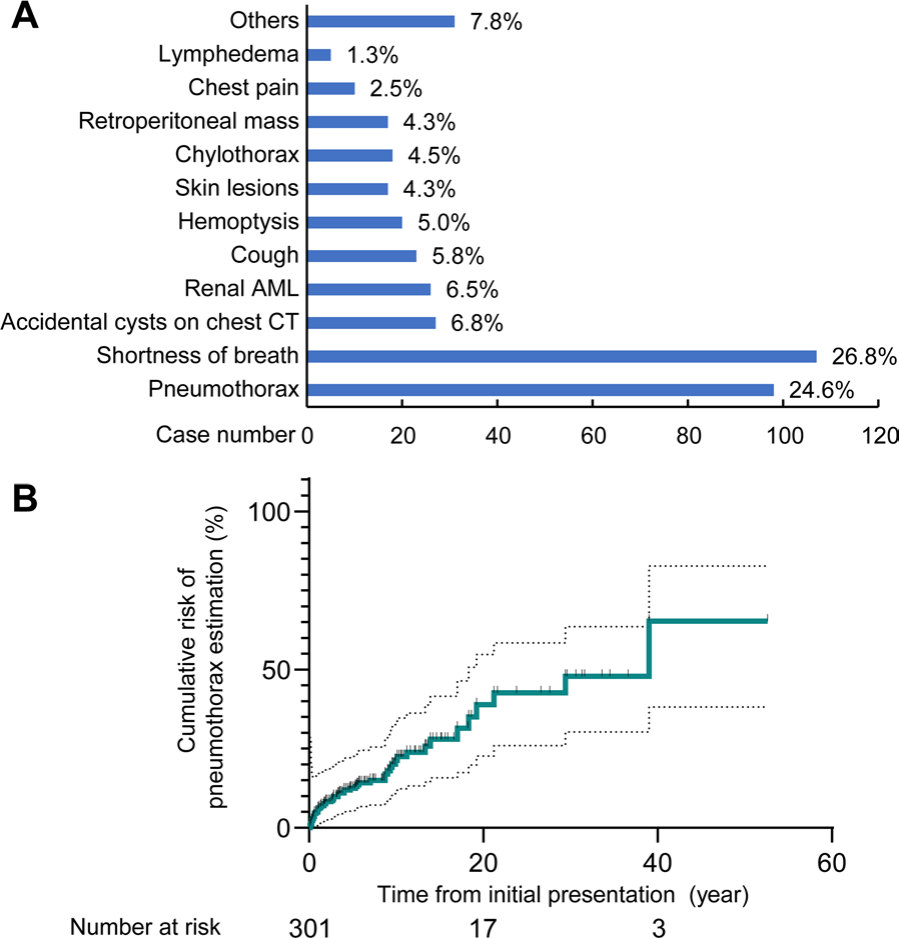
Initial presentation and the cumulative risk of pneumothorax in LAM. **A** Initial chief complaint of LAM patients. Ninety-eight (24.5%) patients had pneumothorax as the first symptom of LAM. Skin lesions refer to skin manifestations in patients with TSC-LAM. **B** In situations where pneumothorax was not the initial symptom of LAM, the cumulative risk of pneumothorax at 5, 10, and 20 years after the initial symptom was 12.5%, 22.5%, and 42.7%, respectively

**Fig. 2:**
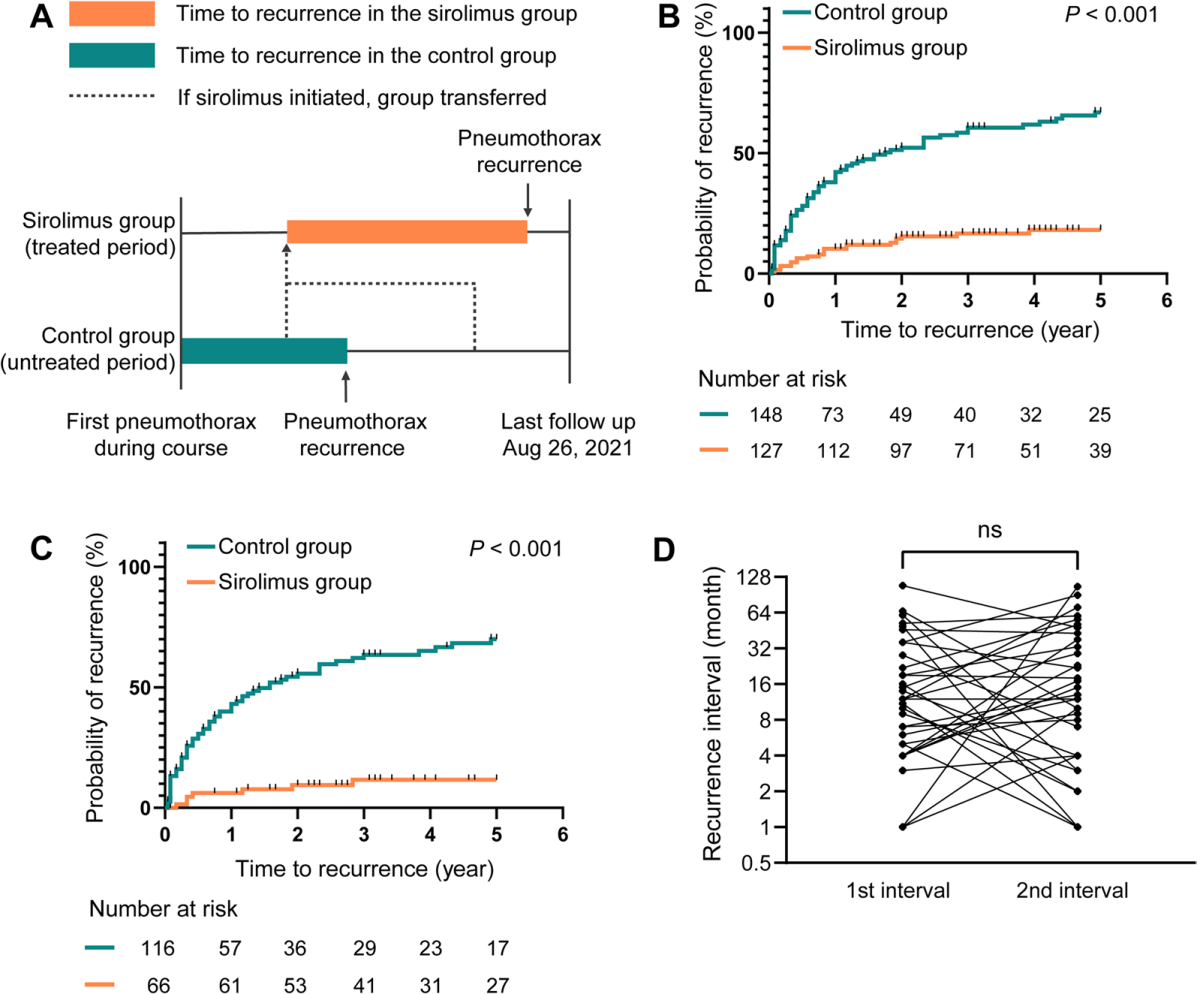
Effects of sirolimus on pneumothorax recurrence. **A** Definition of the sirolimus group (treated period) and control group (untreated period) in the historical prospective self-controlled study. Patients started in the control group, and once they started taking sirolimus, they were transferred to the sirolimus group for continued observation. **B** Kaplan–Meier analysis for the self-controlled study. The 5-year probability of pneumothorax recurrence was 80% lower in the sirolimus group than in the control group (*P* < 0.001 by log-rank test). **C** Kaplan–Meier analysis of LAM patients, removing the influence of pleurodesis. The 5-year risk of pneumothorax recurrence remained lower in the sirolimus group than in the control group (*P* < 0.001 by log-rank test). **D** Comparison of the recurrence interval between the first and the second pneumothorax and between the second and third pneumothorax in LAM patients

To assess whether sirolimus reduced the recurrence risk of pneumothorax in patients with LAM, we performed a Kaplan–Meier analysis in the historical prospective self-controlled study. Depending on whether the patient was taking sirolimus or not, the patients during the untreated period were assigned as the control group and the treated period as the sirolimus group (self-control design). More specifically, LAM patients with a history of at least one pneumothorax were included in this study during the course and follow-up. Patients with a history of sirolimus treatment within one year before the occurrence of the first pneumothorax were excluded from this study (Fig. 1). Untreated patients were initially assigned to the control group, and once patients started sirolimus treatment, they were transferred to the sirolimus group for continued observation (Fig. 2A). The starting point of the control group was defined as the time of the first pneumothorax, and the endpoint was the time of the first pneumothorax recurrence. For the sirolimus group, the starting point was set as the time when sirolimus was initiated, and the endpoint was the time of the first pneumothorax while on sirolimus. By the last follow-up, patients were defined as censored if no outcome event had occurred or, for patients in the control group, if they had started to take sirolimus. Death or loss to follow-up was also defined as a censoring event. The two groups were compared by the log-rank test.

## Results

### Patient characteristics at registration

Of the 455 patients enrolled in the registry study, 402 met the criteria for a confirmed diagnosis of LAM. Three patients were excluded from the analysis because of iatrogenic pneumothorax secondary to transbronchial lung biopsy. Of the 399 patients analyzed, all were female. There were 141 (35.3%) patients with a history of at least one pneumothorax at registration. The median number of pneumothorax occurrences in these patients was 2 (1–4, lower quartile to upper quartile), and 63.1% of patients experienced recurrent pneumothorax. Bilateral pneumothorax was observed in 48.9% of patients with a pneumothorax history, while 28.4% of patients with unilateral pneumothorax were right-sided and 22.7% were left-sided.

The characteristics of patients at registration are presented in Table 1. Of the 399 patients in our study, concomitant diseases at registration are as follows: hypertension (34), diabetes [6], hypothyroidism [5], history of hepatitis B virus infection [5], coronary artery disease [5], Hashimoto's thyroiditis [3], pulmonary tuberculosis [3], chronic kidney disease [2], malignant tumors (2, thyroid cancer and ovarian cancer), and connective tissue diseases (4, Sjogren syndrome, SAPHO syndrome, systemic lupus erythematosus, and Behcet's disease). A total of 36 patients received home oxygen therapy, and 5 patients received progestational hormonal treatment due to menstrual dysfunction.

**Table 1:** Characteristics of patients with LAM at registry

	Without Pneumothorax (N = 258)	With Pneumothorax (N = 141)	*P* value
Age at presentation (year)	37.5 (30.1–43.7)	29.5 (24.3–38.6)	< 0.001
Age at diagnosis (year)	40.0 (33–47)	30.0 (28.5–43)	< 0.001
Age at registration (year)	41(35–48)	36(31–45)	< 0.001
Duration of course (year)	2.3 (0.5–6.2)	5.4 (2.2–9.8)	< 0.001
Body mass index (kg/m^2^)	21.3 (19.5–23.6)	21.1 (19.5–23.0)	0.517
Smoking history	5 (1.9)	5 (3.5)	0.334
Postmenopausal status ^a^	47 (18.2)	4 (2.8)	< 0.001
TSC-LAM	35 (13.6)	19 (13.5)	1.000
Renal angiomyolipoma history	98 (38.0)	61 (43.3)	0.336
Chylothorax history	41 (16.2)	28 (19.9)	0.407
Retroperitoneal mass history	54 (23.5)	23 (18.9)	0.345
Serum VEGF-D (pg/ml)	1948 (957–3385)	1720 (900–3147)	0.502
CT grade ^b^			0.004 ^c^
Grade I	54 (21.0)	17 (12.1)	
Grade II	48 (18.7)	18 (12.9)	
Grade III	155 (60.3)	105 (75.0)	
FEV_1%_ predicted (%)	80.4 (55.0–93.0) N = 223	62.4 (47.6–78.7) N = 94	< 0.001
FVC% predicted (%)	97.9 (88.5–108.3) N = 223	85.7 (74.9–100.2) N = 94	< 0.001
DLCO% predicted (%)	52.6 (33.2–72.9) N = 211	43.9 (35–61.5) N = 88	0.022
6MWT distance (meter)	499.5 (454.5–544.3)	489.0 (432.0–535.0)	0.207

Data are presented as median (lower quartile—upper quartile) or number (percentage). When comparing two groups, the Mann–Whitney test was applied for continuous variables. Fisher's exact test was selected for categorical variables unless specifically noted. TSC-LAM: tuberous sclerosis-associated LAM. VEGF-D: vascular endothelial growth factor; FEV_1_: forced expiratory volume in one second. FVC: forced vital capacity; DLCO: carbon monoxide diffusion capacity; 6MWT: 6-min walk test

^a^ For patients with pneumothorax history, postmenopause was defined as having reached menopause before their first pneumothorax

^b^ Chest CT data were missing in 2 patients, one of them was postmenopausal status

^c^ Calculated by the Cochran–Armitage trend test

Patients with a pneumothorax history had a younger median age at registration (36 years vs. 41 years, *P* < 0.001) and age at diagnosis (30 years vs. 40 years, *P* < 0.001) than patients without a pneumothorax history. In addition, patients with a history of pneumothorax had a higher chest CT grade (Cochran–Armitage trend test, *P* = 0.004) and worse pulmonary function (62.4% vs. 80.4% for FEV_1_% predicted, *P* < 0.001; 85.7% vs. 97.9% for FVC% predicted,* P* < 0.001; 43.9% vs. 52.6% for DLCO% predicted, *P* = 0.022). However, the percentage of patients with missing values was 37% for pulmonary function parameters among patients with a history of pneumothorax. Patients with pneumothorax were less likely to experience menopause before their first pneumothorax (2.8% vs. 18.2%, *P* < 0.001). There were no statistically significant differences in the proportion of TSC-LAM diagnostic category, history of smoking, renal angiomyolipoma, chylothorax, or retroperitoneal mass between patients with and without pneumothorax. Patients with and without pneumothorax had similar body mass index (BMI) and serum VEGF-D.

### Incidence and risk of pneumothorax

Among the 399 patients, three were not followed until August 26, 2021 because of death or loss to follow-up. The mean follow-up duration of the patients was 8.7 years, with a cumulative follow-up of 3468 person-years. During the observation period, 455 pneumothorax events occurred, yielding a calculated annual incidence of 0.13 per person-year.

Ninety-eight (24.6%) patients had pneumothorax as the first symptom of LAM (Fig. 1A). In the remaining patients, the cumulative risk for the development of the first pneumothorax was determined by the Kaplan–Meier method (Fig. 1B). The cumulative risk of developing the first pneumothorax increased gradually with the duration of the disease. The median interval from initial presentation to first pneumothorax was 5.3 years. If pneumothorax was not the first symptom of LAM, the risk of pneumothorax at 5, 10, and 20 years after presentation was 12.5%, 22.5%, and 42.7%, respectively.

### Factors associated with pneumothorax

Age at presentation, postmenopausal status, and CT grade at registration were included in the multivariate logistic regression model (Table 2). Patients with an age at presentation ≤ 35 years had a higher risk of developing pneumothorax (odds ratio = 2.60, 95% CI 1.63–4.15, *P* < 0.001). Patients who were menopausal had a lower risk of developing pneumothorax (odds ratio = 0.21, 95% CI 0.07–0.61, *P* = 0.004). A grade III chest CT was associated with a higher risk of pneumothorax than a grade I chest CT (odds ratio = 2.80, 95% CI 1.50–5.23, *P* = 0.001).

**Table 2:** Multivariate logistic regression of factors associated with pneumothorax

Variable	Case number	Odds ratio (95% CI) ^a^	*P* value
*Age at presentation (y)*			
> 35	194	Reference	-
≤ 35	203	2.60 (1.63–4.15)	< 0.001
*Postmenopausal status*			
No	347	Reference	-
Yes	50	0.21 (0.07–0.61)	0.004
*CT grade*			
Grade I	71	Reference	-
Grade II	66	1.47 (0.66–3.26)	0.344
Grade III	260	2.80 (1.50–5.23)	0.001

Age at presentation, postmenopausal status, and CT grade at registration were included in the multivariate logistic regression model

^a^ Odds ratio for pneumothorax

### Efficacy of sirolimus

In the historical prospective self-controlled study, 152 patients developed pneumothorax. Four were excluded because they were already taking sirolimus before the first pneumothorax. Of the 148 patients in the control group, 127 shifted to the sirolimus group because they started taking sirolimus. The median time from the first pneumothorax to the initiation of sirolimus was 3 (0.6–6.2, lower quartile—upper quartile) years. An initial dose of 1 or 2 mg sirolimus per day was administered, depending on the patient’s body weight, and the dosage was adjusted according to the monitored blood sirolimus valley concentration with a target concentration range of 4–10 ng/ml. Discontinuation of sirolimus for more than 3 consecutive months in a year was considered discontinuation, and no patients in the sirolimus group discontinued treatment. The median number of pneumothorax events that had occurred at the initiation of sirolimus therapy was 2 [1–4].

Pneumothorax recurrence events occurred in 92 (62.2%) patients in the control group and 22 (17.3%) patients in the sirolimus group. The median time to pneumothorax recurrence was 1.75 years in the control group and could not be estimated in the sirolimus group. The results showed that among these 127 patients in the sirolimus group, the total number of pneumothorax events within 2 years prior to sirolimus treatment was 178 and the number of events within 2 years after treatment was 28. The cumulative follow-up was 254 person-year before and 248 person-year after sirolimus treatment. The annual pneumothorax incidence was calculated to be 0.70 per person-year and 0.11 per person-year for 2 years before and after sirolimus treatment, respectively. The 5-year probability of pneumothorax recurrence was 80% lower in the sirolimus group than in the control group (hazard ratio for pneumothorax recurrence, 0.20; 95% CI, 0.14 to 0.30, *P* < 0.001 by log-rank test) (Fig. 2B).

To exclude the effect of pleurodesis on pneumothorax recurrence, thirty-two patients who had pleurodesis at the time of their first pneumothorax in the control group and sixty-one patients with a history of pleurodesis before sirolimus administration in the sirolimus group were excluded. The 5-year risk of pneumothorax recurrence remained lower in the sirolimus group than in the control group (hazard ratio for pneumothorax recurrence, 0.12; 95% CI, 0.07 to 0.18, *P* < 0.001 by log-rank test) (Fig. 2C).

To alleviate the potential effect of time-varying differences caused by the nonparallel study design on the recurrence interval, we performed a pairwise comparison of the first pneumothorax recurrence interval (time from first to second pneumothorax) and the second pneumothorax recurrence interval (time from second to third pneumothorax) in the 34 LAM patients who had three or more pneumothorax episodes. There was no significant difference in the two recurrence intervals (median difference, 2 months, *P* = 0.494) (Fig. 2D).

## Discussion

In our study, approximately one-third of LAM patients had a history of pneumothorax at registration, which is lower than the rates reported in other studies [2–6]. We report that the cumulative risk of the first pneumothorax in patients with LAM increases with time since diagnosis, implying that the proportion of patients with pneumothorax may vary over time. The annual incidence of pneumothorax in patients with LAM in this study was 0.13 per person-year, which was consistent with the incidence rate of 0.12 per person-year found by Cynthia Gonano et al. [7].

In the multivariate logistic regression model, age at presentation ≤ 35 years was a risk factor for the development of pneumothorax in patients with LAM. One explanation is that younger age at presentation is associated with a longer disease course at study registration. High levels of estrogen and progesterone may exacerbate lymphangioleiomyomatosis [12–16]. Thus, the risk factor of age at presentation ≤ 35 years can also be explained by the change in female hormones when estrogen and progesterone levels gradually reach a peak at approximately 25–30 years of age [17, 18]. Postmenopausal status is a protective factor against pneumothorax in LAM patients, which further suggests that estrogen and progesterone play a role in the development of LAM pneumothorax. A higher CT grade indicates more severe cystic lesions in the lungs. It is reasonable that CT grade III would be associated with an increased risk of pneumothorax.

Our data did not show significant differences in VEGF-D level or TSC-LAM composition ratio between patients with and without a history of pneumothorax at registration. Young et al. reported that patients with lower levels of VEGF-D had pneumothorax more frequently than those without such a history [19]. Radzikowska et al. did not find a correlation between serum VEGF-D level and pneumothorax but showed that patients with TSC/LAM had fewer pneumothoraxes and that smoking history was associated with a higher risk of pneumothorax [20]. The clinical characteristics of the pneumothorax and non-pneumothorax groups in those two studies were not described, so there was a possibility that the findings were influenced by factors associated with VEGF-D levels, such as CT grade, TSC-LAM, and lymphatic involvement [20–22]. The percentage of our patients with a smoking history was very low (less than 3.5%), so it was very difficult to compare the relationship between smoking and pneumothorax.

Studies of the relationship between pleurodesis and pneumothorax in LAM are few, though pleurodesis has been recommended for assessment by the ATS/JRS guidelines for LAM patients with pneumothorax [8]. Crude estimates have shown that pleurodesis reduced the risk of pneumothorax recurrence by only 30–45% [8]. We designed a historical prospective self-controlled study to assess whether sirolimus reduced the risk of pneumothorax recurrence. It showed that sirolimus treatment reduced the 5-year risk of pneumothorax recurrence in patients with LAM by more than 80%, regardless of whether the patient had undergone surgical treatment. This suggests that the therapeutic benefit of sirolimus on pneumothorax recurrence in patients with LAM may have been neglected.

Based on the current guidelines, many LAM patients undergo pleurodesis after pneumothorax. Sirolimus therapy is rarely initiated just for a first pneumothorax. Therefore, it is difficult to design a perfect parallel cohort study. In our study, approximately 85% of patients in the control group were finally transferred to the sirolimus group, allowing a self-control setup. We set the starting point for patients in the sirolimus group as the time of initiation of sirolimus rather than the time of the last pneumothorax before sirolimus, mainly to avoid patient recall bias of the time of the last pneumothorax. In this case, the actual recurrence interval (from previous pneumothorax to recurrence) was longer than the time to recurrence defined by our study (from sirolimus to recurrence) in the sirolimus group. Combined with the sirolimus group having a longer time to recurrence than the control group, this finding supports the conclusion that sirolimus has a good ability to prolong the pneumothorax recurrence interval.

Patients in the control group were always earlier than those in the sirolimus group due to the study design, so time-varying confounding remains a potential issue. Firstly, patients in severer status were more likely to receive sirolimus therapy, and a severer condition was associated with a higher pneumothorax risk as we showed earlier. Even with this against factor, our data have shown that sirolimus reduces the risk of recurrence in LAM patients with pneumothorax. Secondly, for patients with multiple pneumothoraxes, especially those receiving closed drainage of pleural cavity, we had concerns about spontaneous "pleurodesis effect" due to repeating pleural irritation. But it was reassuring that there was no significant difference between the first and second recurrence intervals in our patients who had three or more pneumothorax episodes. It was difficult to form a perfect control group, though we have corrected for some confounding factors as much as possible. Parallel controlled clinical trials need to be conducted.

## Conclusion

High CT grade and age at presentation ≤ 35 years were factors associated with an increased risk of pneumothorax in patients with LAM. Postmenopausal status was associated with a reduced risk of pneumothorax. Sirolimus significantly reduces the risk of pneumothorax recurrence in patients with LAM. Whether pneumothorax events should trigger sirolimus therapy requires further investigation.

## Data Availability

The datasets used and analyzed during the current study are available from the corresponding author on reasonable request.
